# Inhibition of platin-induced BCL2 increase overcomes chemoresistance in squamous cell carcinoma of the head and neck through resensitization to cell death

**DOI:** 10.1016/j.tranon.2025.102308

**Published:** 2025-02-18

**Authors:** Anne-Sophie Becker, Friederike Klauk, Thomas Freitag, Daniel Fabian Strüder, Björn Schneider, Annette Zimpfer, Claudia Maletzki

**Affiliations:** a Institute of Pathology, Rostock Medical Center, Germany; b Hematology, Oncology, Palliative Medicine, Department of Medicine, Clinic III, Rostock University Medical Center, Germany; c Department of Otorhinolaryngology, Head and Neck Surgery “Otto Koerner”, Rostock Medical Center, Germany

**Keywords:** Head and neck squamous cell carcinoma, BCL2, ABT-199, Cisplatin, Chemoresistance, Apoptosis, Autophagy

## Abstract

The clinical outcome of head and neck squamous cell carcinoma (HNSCC) remains poor with high recurrence rates, in part due to resistance to concurrent platinum-based chemotherapy. The anti-apoptotic BCL2 protein is involved in apoptosis resistance and tumor cell invasion/migration. Here, we test whether BCL2 overexpression predicts poor therapeutic response of HNSCC to cisplatin-based chemoradiotherapy and the effects of selective BCL2 inhibition on cisplatin-induced cell changes *in vitro.*

BCL2 immunostatus was correlated with survival after chemoradiotherapy in a uniformly treated HNSCC cohort. The combination therapy of ABT-199, a BCL2 inhibitor, and cisplatin was evaluated *in vitro* using corresponding patient-derived cell lines. Colony formation and the mode of cell death were analyzed in-depth.

Patients with BCL2-positive tumors (44/254) prior to treatment (either radiation, cisplatin monotherapy, or both) had shorter overall and progression-free survival (log-rank; *p* = 0.048) and a higher rate of tumor relapse (Fisher's exact test; *p* = 0.0032). BCL2 inhibition alone had no effect on cell functions in our triple panel of cisplatin-sensitive cell lines but enhanced cisplatin-induced effects. Rates of autophagy and cell death, including methuosis, were doubled, while epithelial-mesenchymal transformation was inhibited.

As selective inhibition of BCL2 is available and standard of care in other malignancies, its immunohistochemical assessment could help personalize therapy by identifying a subpopulation to overcome chemoresistance, particularly in locally advanced HNSCC.

## Highlights

• Cisplatin resistance is common in head and neck squamous cell carcinoma (HNSCC) and may be mediated by anti-apoptotic pathways, including Bcl-2, which is not specifically targeted in routine patient care.
• BCL2 inhibition by the selective BCL2 inhibitor ABT-199 (=venetoclax) alone had no effect on cell functions in a triple panel of cisplatin-sensitive cell lines but enhanced cisplatin-induced effects.
• Rates of autophagy and cell death, including methuosis (a form of non-apoptotic cell death), were doubled, while epithelial-mesenchymal transformation was inhibited.
• In a retrospective HNSCC cohort, pre-treatment BCL2-positive tumors have shorter progression-free and overall survival and higher tumor recurrence rates when BCL2 status is analyzed by immunohistochemistry.
• Adding selective inhibition of BCL2 to standard care may help to overcome chemoresistance in (locally advanced) HNSCC, where treatment options are limited.

## Graphical abstract

**Figure:**
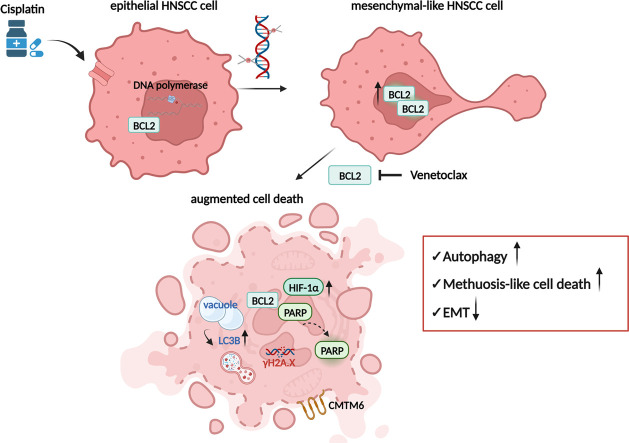

## Introduction

Head and neck cancers are a heterogeneous group of malignancies with increasing incidence worldwide. The majority of cancers are squamous cell carcinomas arising from the epithelium of the oral and nasal cavities, pharynx, and larynx [1]. Existing treatments can improve survival rates, but serious consequences can occur, including disruption of vital anatomy, aesthetic impairment, and associated psychosocial problems [2].

Chemotherapy is an essential component of multimodal treatment strategies for HNSCC. Approximately 60 % of patients with advanced disease receive either curative or palliative chemotherapy during treatment [3]. Depending on clinical presentation, radiotherapy may be combined with chemotherapy, which is mainly cisplatin [1]. Cisplatin crosslinks DNA in several ways, interfering with cell division through mitosis. The damaged DNA triggers DNA repair mechanisms, which in turn activate apoptosis when repair proves impossible [4]. This tumor cell death is counteracted by resistance mechanisms, which is a limitation to treatment options [5]. The avoidance of therapy-induced apoptosis is a hallmark of acquired resistance. Inhibition of apoptosis is considered an essential step in the transition of normal to malignant cells. Among the key regulators of apoptosis, the B-cell lymphoma 2 (BCL2) protein family plays an important role.

BCL2 is an anti-apoptotic protein and is normally not upregulated in HNSCC. Aberrant overexpression occurs in approximately 15–25 % of HNSCC tumors, resulting in resistance to cisplatin-induced apoptosis [6,7]. A substantial body of research shows that targeting BCL2 family members has synergistic activity with standard therapies that use cytotoxic agents such as cisplatin to kill HNSCC cells [8,9]. While preclinical studies were promising, translation into clinical practice was not feasible due to poor oral absorption or toxicity related side effects [10]. ABT-199 (= venetoclax), which exclusively targets the BCL2 protein, is EMA/FDA-approved for the treatment of several types of leukemia [11,12]. Venetoclax re-sensitized leukemic tumors towards chemotherapy [13]. In combination to other drugs, the selective inhibition of BCL2 by venetoclax mediated chemotherapeutic effects in breast, cervical and colon cancer as well as in osteosarcoma ([[14], [15], [16]]. While the pan-BCL-inhibitor AT-101 is a competent enhancer of radiation-induced apoptosis in HNSCC *in vitro* [17], venetoclax has not been studied in HNSCC patients receiving cisplatin. Venetoclax in combination with radiation  and the anti-EGFR antibody cetuximab enhanced antitumoral effects in HNSCC cells, including inhibition of proliferation, invasion/migration, and overcoming apoptosis resistance [7].

The aim of this study is to evaluate the potential of venetoclax as a chemotherapeutic sensitizer, as (radio)chemotherapy is a fundamental part of treatment strategies in HNSCC. Therefore, we investigated the effects of combined venetoclax cisplatin treatment on cellular expression patterns and cytotoxic effects in patient-derived HNSCC cell lines. We also analyzed BCL2 protein expression in HNSCC samples as a potential therapeutic option.

## Material and methods

### Patient cohort

Pretreatment samples from locally advanced or recurrent/metastatic HNSCC patients, diagnosed between 2000 and 2021, receiving either cisplatin, radiation or radiochemotherapy as first line adjuvant treatment were collected from Rostock Pathology archives. Patients’ characteristics including HPV status and genomic alterations were obtained from clinical records and pathology reports. Status of PD-L1, CMTM6 and density of tumor infiltrating lymphocytes (TIL) were extracted from prior studies [18]. Using pseudonyms, follow-up data were obtained from the regional Cancer Registries. Tumor tissue was obtained from formalin-fixed, paraffin-embedded (FFPE) specimens. Tissue microarrays (TMAs) of three representative areas were constructed using 1 mm cores with a manual tissue microarrayer (Beecher Instruments, Silver Spring, MD, USA). Tissues were collected with patient consent. The institutional ethic committee at the University Hospital Rostock approved to the study (A2018–0003; A2022–0120) which was conducted in accordance with the Declaration of Helsinki of 1975.

### Immunohistochemistry

Slides of 4 μm were used. Heat-induced antigen retrieval was performed with a high pH buffer (20 min at 97 °C). The following steps were performed in an Autostainer link 48 instrument (Dako, Hamburg, Germany): 5 min of incubation in peroxidase-blocking buffer followed by 20 min of incubation with primary antibody (BCL2: 1:100, SC06–86, GeneTex, Alton Pkwy Irvine, CA, USA) and 3,3′- diaminobenzidine (DAB) detection using the Dako-kit K8000 according to the instructions of the manufacturer. Slides were counterstained with hematoxylin. Lymphocytes served as positive controls.

Tumor cells with membranous and/ or cytoplasmic BCL2 staining of any intensity were counted manually in the 400x- magnification, and scoring was based on the percent of positive cells as negative (<29 %) or positive (≥30 %).

### Cell culture

The HNSCC 16 (HPV*^neg^/* p16*^neg^*), 46 (HPV*^neg^/* p16*^pos^*) and 48 (HPV*^neg^/* p16*^neg^*) cell lines were derived from patient xenograft models and established by our group [19]. Cells’ characteristics including detailed molecular profiling and chemosensitivity have been described previously [19]. Cells were cultured in DMEM supplemented with 10 % fetal bovine serum (FBS), 100 U/mL penicillin, 0.1 g/L streptomycin, and 6 mM l-Glutamine.

### Colony formation assay

Cells were seeded in 24-well plates at 500 cells/well. After 24 h, cells were treated for 2 × 72 h with cisplatin (University Hospital Rostock Pharmacy), venetoclax (MedChemExpress, NJ, USA), or a combination of both at doses determined before: abemaciclib [HNSCC16 P1 M1/HNSCC46 P0 M2: 1 µM, HNSCC48 P0 M1: 0. 1 µM], cisplatin [HNSCC16 P1 M1/HNSCC46 P0 M2: 1 µg/mL, HNSCC48 P0 M1: 0.05 µg/mL]. Readout was performed using crystal violet staining. Colonies were counted with ImageJ (Hersteller). The Bliss Independence model was used to analyze potential synergistic and additive effects between the compounds.

### RNA isolation, cDNA synthesis, and quantitative real-time PCR

Total RNA was isolated with RNeasy Mini Kit (Qiagen, Hilden, Germany) according to the manufacturer's instructions. RNA was reverse-transcribed into cDNA from 1 µg RNA using 1 µl dNTP mix (10 mM), oligo (dT)15 primer (50 ng/µl), 1 µl reverse transcriptase (100 U) and 4 µl 5x RT buffer complete (all purchased from Bioron GmbH, Ludwigshafen, Germany). cDNA synthesis conditions were as follows: 70 °C for 10 min, 45 °C for 120 min, 70 °C for 10 min. Target cDNA levels of HNSCC cell lines were analyzed by quantitative real-time PCR using TaqMan Universal PCR Master Mix (Thermo Fisher Scientific, Darmstadt, Germany) and predesigned BCL2 TaqMan gene expression assay labeled with 6-FAM-3′ BHQ-1: Hs00608023_m1 (Thermo Fisher Scientific). GAPDH was used as housekeeping gene. Primers were self-designed (Hersteller) 5′ HEX BHQ-TGCCATCAATGACCCCTTCATTG-3′; for: 5`-TCACCAGGGCTGCTTTTAAC-3`; rev: 5`-GGGTGGAATCATATTGGAACA-3`: Reaction was performed 12.5 ng cDNA with the following PCR conditions: 95 °C for 10 min, 40 cycles of 15 s at 95 °C, and 1 min at 60 °C. All reactions were run in triplicates. The mRNA levels of target genes were normalized to GAPDH. The general expression level of each sample was considered by calculating 2-ΔΔCT resulting from the difference between ΔCT_target_-ΔCT_Calibrator_. Untreated cells were used as calibrator.

### Flow cytometry

Apoptotic/necrotic cells were detected by Yo-Pro-1 and propidium iodide (PI) staining as described before [20]. Briefly, early and late apoptotic cells were detected by either Yo-Pro-1 or Yo-Pro-1/propidium iodide (PI) positivity. Necrotic cells were defined as Yo-Pro-1 negative/PI positive.

Extended flow cytometry included assessment of BCL2 (APC REA anti-human BCL, please see below for details), as well as several cell death and DNA-damage associated markers. Therefore, two in-house panels were applied. Panel 1 and 2 were used to study apoptosis, DNA damage, autophagy, cell stress, and methuosis. Panel 3 was used to identify epithelial-mesenchymal transition (EMT) and glucose transporters. For this purpose, 0.5 × 10^6^ cells per panel were taken and processed. All procedures were performed using staining buffer (PBS, 2 mM EDTA, 2 % BSA).

Panel 1: Extracellular staining was done using Apotracker green (Stock: 80 µM, 1:400 final, Biolegend, San Diego, USA), CYTO-ID (0.5 µl/sample, CYTO-ID® Autophagy detection kit 2.0, Enzo Life Sciences GmbH, Lörrach, Germany). Membrane permeabilization was done as first step (True Nuclear Transcription Factor Buffer Set, Biolegend, True-Nuclear™ 1X Fix concentrate, 45 min, RT). Then, the True-Nuclear™ 1X Perm Buffer (Biolegend) was added, cells were washed (350 x g, 5 min) and stained with antibodies for intracellular staining (in 100 µl True-Nuclear™ 1X Perm Buffer): Alexa Fluor 700 mouse-anti-human cleaved PARP (Clone F21–852, BD Biosciences, Heidelberg, Germany), PE/Cyanine7 mouse anti-H2A.X phospho (clone: 2F3, 1:40, Biolegend), V450 rat anti-histone H3 (1:40, BD Biosciences), and Alexa Fluor 647 rabbit-anti-human LC3B (clone: 1251A, R&D Systems, Minneapolis, USA). Staining was done for 30 min at RT, reaction was stopped with True-Nuclear™ 1X Perm Buffer, followed by two washing steps (350 x g, 5 min). Cells were resuspended in 0.35 ml staining buffer.

Panel 2: Extracellular staining was done for 20 min at RT in staining buffer (in 100 µl): FITC mouse anti-human CD107a (clone: H4A3, Biolegend) and Alexa Fluor 594 rat anti-human Rab7a (clone: W16034A Biolegend). Intracellular staining was done after permeabilization as described for Panel 1 using the following antibodies: APC REA anti-human BCL2 (clone: REA872, Miltenyi Biotec, Bergisch-Gladbach, Germany) and Alexa Fluor 405 mouse anti-human Hif1α (clone: 241,812, R&D). All subsequent steps were done as described above. Cells were resuspended in 0.35 ml staining buffer.

Panel 3: Extracellular staining was done for 20 min at RT in staining buffer (in 100 µl): PE/Cyanine7 mouse anti-human CD325 (1:25, N—Cadherin, clone: 8C11, Biolegend), and APC-Vio 770 mouse anti-human CD324 (1:15, E-Cadherin, clone 67A4, Biolegend). Afterwards, cells were washed two times followed by membrane permeabilization (BD Transcription Factor Buffer Set, BD, 1x Fix/Perm Working solution, 45 min, 4 °C). Then, the 1x Perm/Wash Buffer (BD) was added, cells were washed (350 x g, 5 min) and were stained with antibodies for intracellular staining (in 100 µl 1x Perm/Wash Buffer): Alexa Fluor 647 mouse anti-human Glut1 (1:500, BD) and Alexa Fluor 700 mouse anti-human Glut4 (1:100, clone: # 925,932, R&D). Staining was done for 30 min at room temperature, reaction was stopped with 1x Perm/Wash Buffer, followed by two washing steps (350 x g, 5 min). Cells were finally suspended in 0.35 ml staining buffer.

All measurements were done on a spectral flow cytometer (Cytek Aurora, Cytek Biosciences, Fremond, California, United States) in the Core Facility for Cell Sorting and Cell Analysis, University Medical Center Rostock, Rostock, Germany.

### Immunofluorescence

Cells were fixed with 2 % paraformaldehyde (PFA) w/o methanol (15 min, Thermo Fisher Scientific), permeabilized, and blocked with 0.5 % Triton X-100 (Thermo Fisher Scientific,) in 2 % BSA (PAN-Biotech, Aidenbach, Germany) for 60 min. Slides were incubated with alexa Fluor® 594 anti-Vimentin antibody (clone: O91D3, Biolegend) over night at 4 °C, washed three times with PBS, followed by phalloidin green staining (1:50 in PBS) for 20 min. Nuclei were counterstained with DAPI (1:1.000, Biomol, Hamburg, Germany) and cells were analyzed using a Zeiss microscope Axiovert A1 (Zeiss, Oberkochen, Germany).

### Statistics

All values are given as mean ± SD (*in vitro* analysis) or mean ± SEM (*in vivo* approach). Statistical evaluation was performed using GraphPad PRISM software, version 8.0.2 (GraphPad Software, San Diego, CA, USA). The criterion for significance was set to *p* < 0.05. *In vitro* experiments were executed in biological triplicates and summarized as mean values with standard deviation. After proving the assumption of normality (Shapiro–Wilk test), one-way ANOVA (Dunnett's multiple comparison), two-way ANOVA (Tukey's multiple comparison) or *t*-test was performed. If the normality test failed, the Kruskal–Wallis or U-Test was performed. Survival distributions were compared using the log-rank test.

## Results

### Patients with HNSCC tumors expressing BCL2 had a higher risk of recurrence

**Fig. 1:**
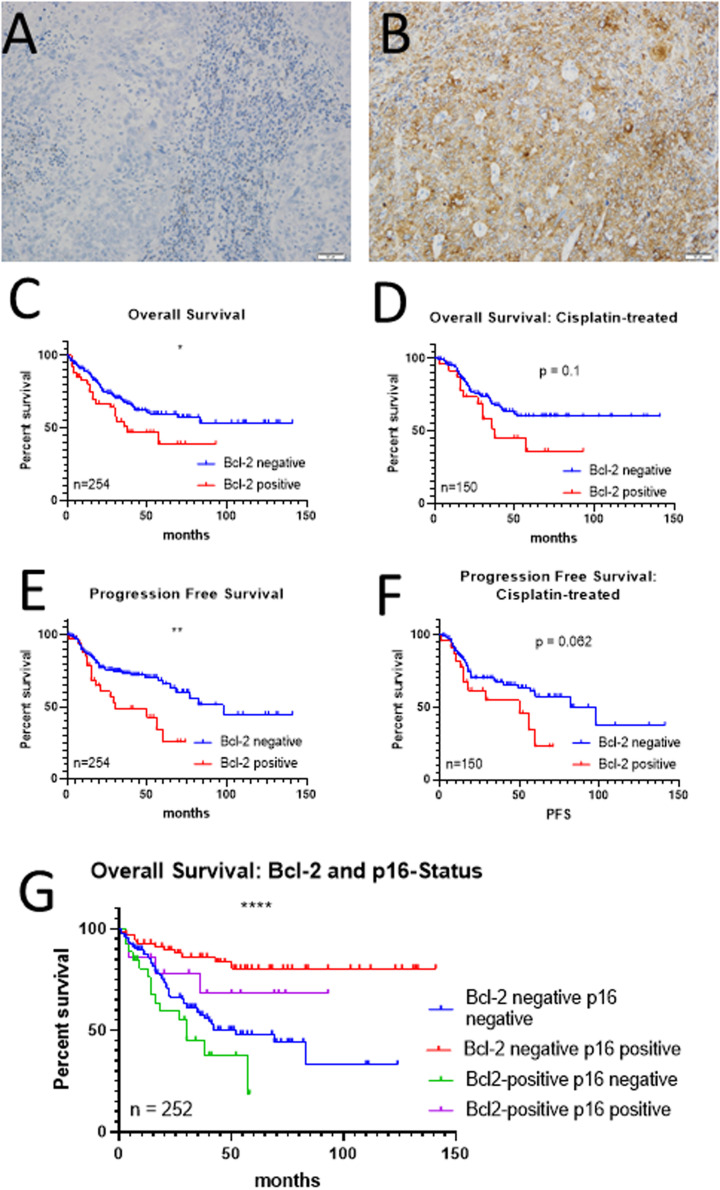
Immunohistochemical staining for BCL2 and its impact on survival: A, B) representative images of a BCL2 negative (A) and positive HNSCC specimen (B), respectively (200x). C-F) Kaplan-Meier plots of overall (C, E) and progression-free (D, F) survival for the whole cohort (receiving cisplatin, radiation, or radiochemotherapy) (C, E) and the Cisplatin-treated subgroup (D, F). BCL2 expression was related to a shortened survival with patients of BCL2-positive, p16^negative^ tumors having the shortest survival (G). * *p* < 0.05; ** *p* < 0.01; **** *p* < 0.0001.

Overall, 17 % of the 254 included HNSCC samples were BCL2 positive (Fig. 1A and B). Details of the representative cohort are shown in suppl. Table 1. 28 % of patients suffered from p16-positive tumors. In BCL2-positive tumors, the staining intensity was homogeneous. Intratumoral heterogeneity, defined as a percentage of labeled tumor cell fractions (35 to 70 %), was observed in 50 % of positive cases. No correlation with PD-L1 expression as assessed by the combined positive score nor its post-translational stabilizer CMTM6 not tumor infiltrating lymphocytes (TILs) was found in cases where data on these markers were available (*n* = 151). The recurrence rate was significantly higher for BCL2 positive tumors compared to the BCL2 negative counterpart (*p* = 0.0032, Fisher´s exact test). Patients whose tumors were BCL2 positive had a significantly shorter OS regardless of treatment (*p* = 0.048; log rank; Fig. 1C). Further stratification revealed that this effect was significant only in patients receiving radiochemotherapy (*n* = 128; *p* = 0.01), but not in the cisplatin- or radiation-treated subgroups (Fig. 1D). Progression-free survival (PFS) was significantly shorter in patients with BCL2 positive tumors (*p* = 0.003) than in the BCL2 negative group. This effect was significant in patients receiving radiation (*n* = 68) or radiochemotherapy (*p* < 0.0001 and *p* = 0.003, respectively), but not in the cisplatin-treated subgroup (Fig. 1E and F).

**Table 1:** Clinicopathological characteristics of the cohort (*n* = 254).

	Bcl-2	negative	Bcl-2	positive	
Characteristic	No Cisplatin	+ Cisplatin	No Cisplatin	+ Cisplatin	*p-*value*
n	88	122	16	28	
Median age (range)	59,5 (36–70)	60 (40–83)	53 (45–67)	54 (46–67)	n.s
Gender			n.s		n.s
male	59	100	16	26	
female	29	22	3	2	
Localization			n.s		n.s
Oral	15	34	5	9	
Oropharyngeal	43	41	8	12	
Hypopharyngeal	8	22	1	2	
Laryngeal	22	24	2	5	
UICC stage					n.s
I/II	48	27	5	8	
III/IV	40	95	11	20	
Treatment					
surgery/radiation only	32	–	6	–	n.s
+ radiation	56	–	10	–	
+ chemotherapy	–	60	–	15	
+ chemoradiation	–	62	–	13	
2-year OS	71 %	57 %	50 %	54 %	n.s
Recurrence					
Yes	20 %	34 %	44 %	54 %	**
P16^#^					n.s
negative	58	85	12	20	
positive	28	37	4	8	

* Significance level: * *p* < 0.05; ** *p* < 0.01; # *n* = 252; n.s, not significant.

We examined whether BCL2 expression correlated with other clinical characteristics to test the hypothesis that BCL2 expression is a marker of poor therapeutic outcome. However, none of the characteristics, including anatomic site and p16 status, differed significantly after stratification according to BCL2 status (Suppl. Table 1).

### BCL2 in cell lines and its treatment-related changes

**Fig. 2:**
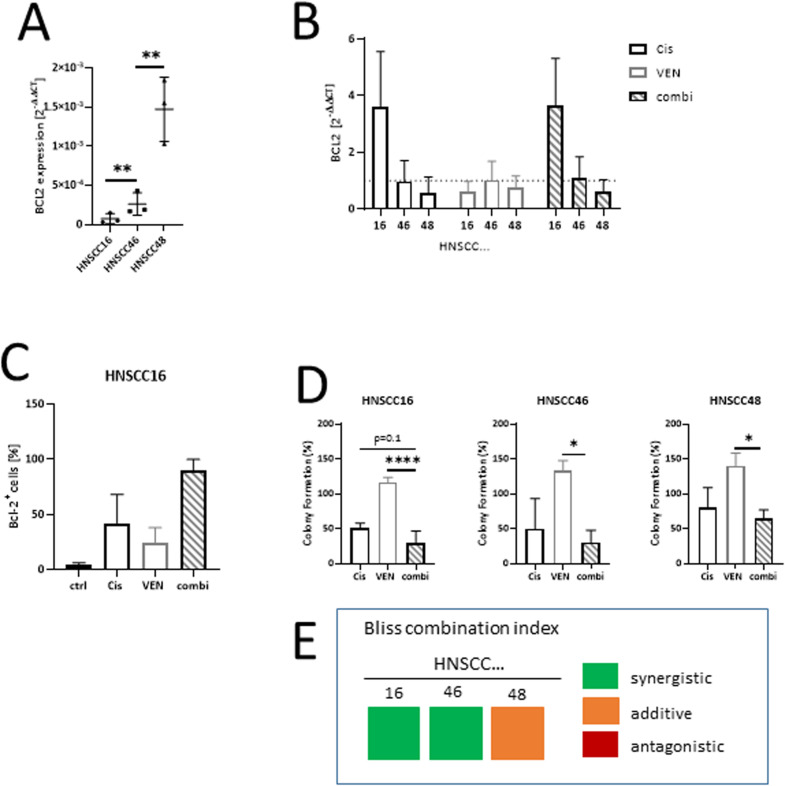
BCL2 status in patient-derived HNSCC cells & impact of treatment on cell growth: (A) Pre-treatment mRNA expression levels with lowest levels in HNSCC16 and highest level in HNSCC48, which was derived from a patient with recurrent disease. ** *p* < 0.01; one-way ANOVA (Tukeys multiple comparison test). (B) Treatment-induced changes in mRNA expression levels. (A, B) mean ± SD, *n* = 3 independent experiments. Values are given as 2^-ΔΔCT^. Untreated cells were used as calibrator. (C) Treatment-induced changes in protein levels using HNSCC16. Notably, residual cells in the venetoclax/cisplatin combination group showed highest BCL2 levels (y-axes related to non-treated controls). mean ± SD, *n* = 3 independent experiments. (D) Colony formation assay. While venetoclax monotherapy had no effect on colony formation, the cisplatin induced inhibition doubles in all cell lines when venetoclax and cisplatin are combined (y-axes related to non-treated controls). mean ± SD, *n* = 3 independent experiments. * *p* < 0.05; **** *p* < 0.0001; one-way ANOVA (Tukey's multiple comparison test) (E) Bliss combination index for cisplatin + venetoclax therapy in the three cell lines to calculate synergistic effects of the combination in comparison to either monotherapy.

Three patient-derived cell lines from our HNSCC biobank were included [21,22]. BCL2 expression was confirmed in all three cell lines with, however, with cell line dependent differences (Fig. 2A). Cisplatin treatment increased BCL2 expression levels in 2/3 cases. Like illustrated in Fig. 2B, BCL2 levels rise after cisplatin treatment with the highest increase in cell line HNSCC16, where this anti-apoptotic molecule normally shows low mRNA levels. Venetoclax alone slightly inhibited BCL2 expression in all three cell lines, but this effect is lost in the combination. In BCL2^low^ HNSCC16, treatment with either cisplatin or venetoclax increased the rate of BCL2 positive cells, and the fraction was higher when both drugs were combined (Fig. 2C).

**Fig. 3:**
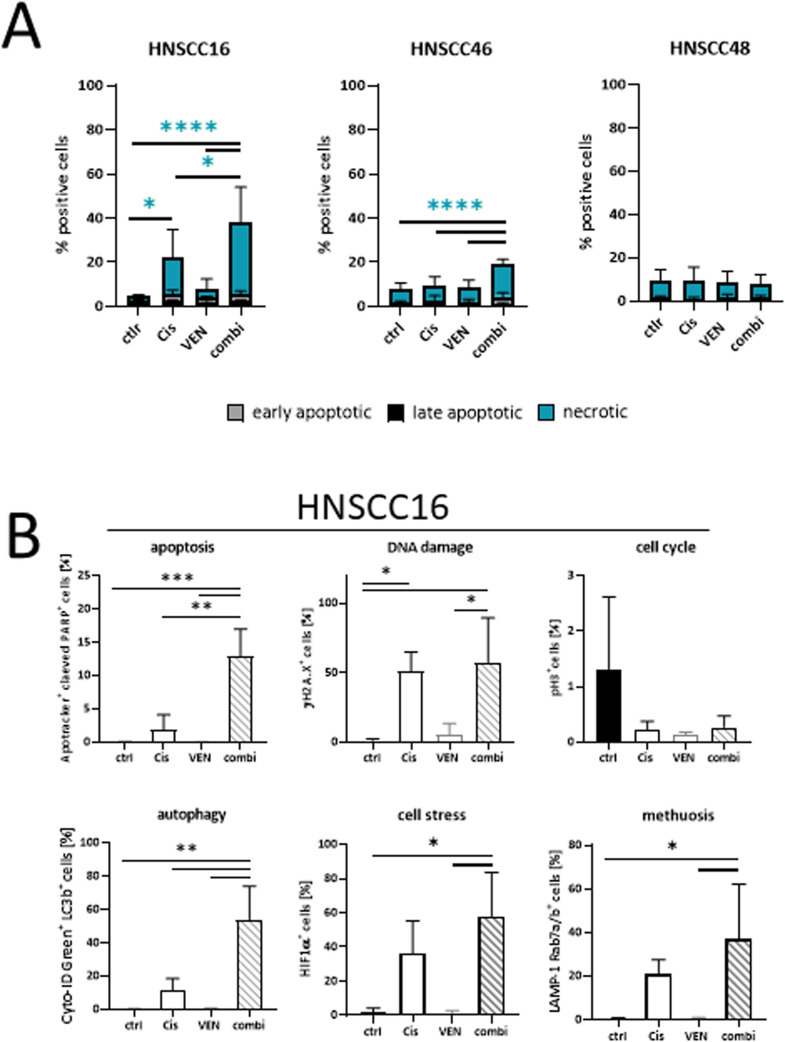
Treatment-related changes in patient-derived HNSCC cells (flow cytometry). (A) Apoptosis/necrosis assay. Cells were stained with Yo-Pro 1 iodide and PI. Cells that were positive for Yo-Pro 1 iodide were defined as early apoptotic, cells that were positive for PI were defined as necrotic, and double positive cells were defined as late apoptotic. Apoptosis/necrosis assay was done after 2 × 72 h. mean ± SD, *n* = 3 independent experiments. * *p* < 0.05; **** *p* < 0.0001; one-way ANOVA (Tukey's multiple comparison test) (B) Changes in apoptosis, DNA damage, cell cycle, autophagy, cell stress, and methuosis are shown for HNSCC16. While cisplatin alone induced apoptosis, DNA damage, autophagy, cell stress, and methuosis by reducing cell cycle at the same time, effects are stronger using double therapy with a significantly increase for cells undergoing apoptosis, autophagy, and methuosis. Venetoclax had no impact on cell death. mean ± SD, *n* = 3 independent experiments. * *p* < 0.05; ** *p* < 0.01; *** *p* < 0.001; one-way ANOVA (Tukey's multiple comparison test).

Then, the effect of either treatment on colony formation was studied (Fig. 2D). While venetoclax alone was not able to reduce colony formation, its combination with cisplatin reinforced this effect in all three cell lines, irrespectively of the pre-existing BCL2 mRNA expression status. Notably, this growth inhibitory effect was synergistic in HNSCC16 and HNSCC46 (Fig. 2E). Subsequent flow cytometric apoptosis/necrosis measurement confirmed antitumoral effects, which were mainly attributable to necrotic cell death (Fig. 3A). While this was highly significant in HNSCC16 and HNSCC46, no such therapeutic effects were detectable in the BCL2^high^ HNSCC48 cells, which were classified as resistant.

Taken together, venetoclax reinforces the cytotoxic effects of cisplatin monotherapy.

### Combination of venetoclax and cisplatin and its effect on the mode of cell death

To gain a deeper understanding of the mechanisms of response and eventually resistance, a full spectrum flow cytometric approach was applied to HNSCC16 cells (Fig. 3B). With this analysis, complex mechanisms of cell death were identified and the effects of cisplatin monotherapy were mostly enhanced by the addition of venetoclax. In detail, we identified significantly more cleaved PARP^+^ apoptotic cells, higher levels of DNA damage (γH2A.*X*^+^), and reduced amounts of cells in G2-phase (pH3^+^). Another rather unexpected finding was the massive induction of autophagy (Cyto-ID^+^LC3b^+^), accompanied by high levels of HIF1αand signs of methuosis. The latter was detected by LAMP-1/Rab7a positivity and the number of cells undergoing this type of cell death almost doubled when both drugs were combined. Thus, the addition of venetoclax to cisplatin enhances the inhibitory and pro-apoptotic effects of both monotherapies *via* the induction of autophagy and methuosis.

### Combination of venetoclax and cisplatin and its effect on epithelial mesenchymal transition

**Fig. 4:**
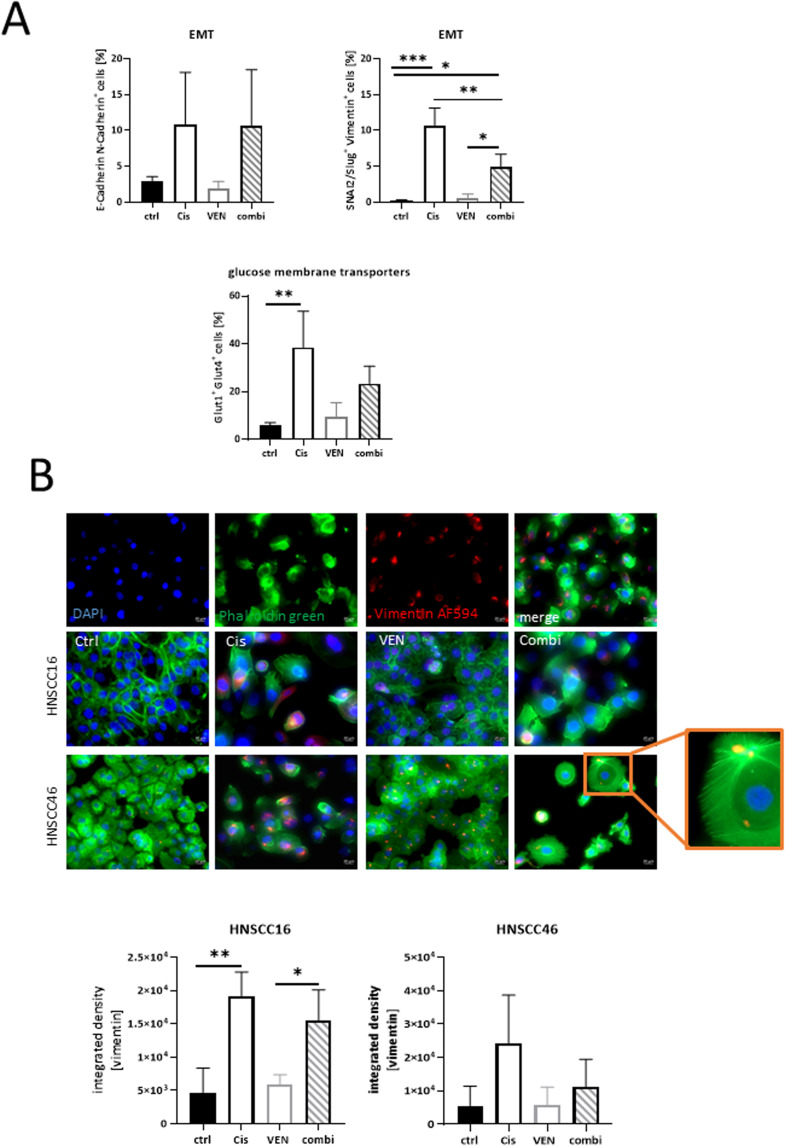
Impact of BCL2 blockade on epithelial-mesenchymal transition (EMT). (A) Flow cytometric staining for EMT markers in HNSCC16. Adding venetoclax to cisplatin has no effect of cisplatin induced changes in the ratio of cells expressing E-Cadherin/N—Cadherin but reduced the ratio of cisplatin monotherapy-induced increased numbers SNAIL^+^/ Vimentin^+^ cells. mean ± SD, *n* = 3 independent experiments. * *p* < 0.05; ** *p* < 0.01; *** *p* < 0.001; one-way ANOVA (Tukey's multiple comparison test). (B) Immunofluorescence to confirm flow cytometry results. Semi-quantitative analysis of EMT marker vimentin was done for HNSCC16 and HNSCC46 cells after treatment. With this analysis, the potent antitumoral effect of the venetoclax/cisplatin combination was confirmed, but had minor influence of vimentin intensity in residual cells. Original magnification 400x. mean ± SD, *n* = 3 independent experiments. * *p* < 0.05; ** *p* < 0.01; one-way ANOVA (Tukey's multiple comparison test).

Epithelial-mesenchymal transition (EMT), *i.e.* the conversion of immobile epithelial cells into motile mesenchymal cells and the alteration of cell–cell adhesion and extracellular matrix is an unwanted but common resistance mechanism after cisplatin-based chemotherapy. To examine if this mechanism may also play a role here, we analyzed the status of different EMT markers. As shown in Fig. 4A, the fraction of E-cadherin^+^/ N—Cadherin^+^cells increased in HNSCC16 cells as a result of cisplatin monotherapy or its combination with venetoclax indicating the so-called “cadherin switch”. The ratio of SNAIL^+^/ Vimentin^+^ cells as another indicator of EMT increased tenfold after cisplatin treatment of BCL2^low^ HNSCC16 cells. Notably, the addition of venetoclax to cisplatin partially reversed this effect by approximately 50 %. This reduction implies a significant inhibition of cisplatin-induced EMT by venetoclax. In support of this, the levels of Glut1^+^/Glut4^+^ cells were lower in the combination, compared to cisplatin monotherapy. Using phalloidin green as an epithelial and vimentin as a mesenchymal marker, this effect is illustrated in Fig. 4B, where vimentin positive cells are markedly reduced in the combination (Fig. 4B). Although this did not reach statistical significance, we would like to mention this positive trend of reversed EMT in both HNSCC16 and HNSCC46 cells. Besides, cells in the combination group showed morphological signs of cell stress (as evidenced by the presence of stress fibers, please see orange box for visualization). Since this was not seen after monotherapy, we conclude that enhanced cell stress is a contributing factor to the increased cell death in the combination.

## Discussion

Our study confirms that HNSCC patients with BCL2-positive tumors have a shorter PFS and OS. We demonstrate a significant inhibition of cell proliferation and an increase in cell death when HNSCC cells are treated with the combination of cisplatin and venetoclax. Inhibition of BCL2 by this drug together with cisplatin reduced EMT capacities in cells, independent of pre-existing BCL2 status. Combined therapy targeting anti-apoptotic pathways, which normally promote chemotherapy resistance, may overcome cisplatin resistance-related tumor escape by re-inducing apoptosis and inhibiting EMT.

BCL2 has been studied in HNSCC for decades [23] with conflicting predictive value. As Michaud et al. conclude in their 2010 study, this can be partly explained by the heterogeneity of collectives and different scoring systems. Using a simple binary scoring system, BCL2 is associated with shorter PFS in patients receiving radio-chemotherapy. This is consistent with the findings of Michaud, who induced BCL2 expression in cell lines using a retroviral expression vector and observed an increased cisplatin resistance. Here, we use venetoclax to inhibit BCL2 activity and see congruent effects. More recently, a French group reported increased tumor cell death *in vitro* and delayed tumor growth with prolonged lifespan in tumor-bearing mice after treatment with venetoclax, cetuximab (an EGFR inhibitor) and radiation. The addition of another potent but less selective BCL2 inhibitor (ABT-263, or navitoclax) also increased tumor cell apoptosis *in vitro* independent of p53 status and significantly delayed tumor growth in mice [9]. The authors concluded a senolytic-mediated clearance of senescent HNSCC cells. In addition to inhibiting BCL2, navitoclax also inhibits its family members Bcl-xL and Bcl-w, which may add to the toxicity for HNSCC patients, reducing its clinical utility [8]. Despite efforts to treat patients with advanced HNSCC, median survival remains poor [1]. Venetoclax in combination with radio-chemotherapy may represent a new therapeutic option, particularly for patients with cisplatin-resistant tumors, and further clinical trials are needed to demonstrate its efficacy [24].

While the poor outcome of BCL2-positive HNSCC patients and the ability of venetoclax to inhibit cell proliferation, invasion/migration, and resistance to apoptosis have been demonstrated, a novel and unique finding of our study is the enhanced cisplatin-mediated cytotoxicity in combination with venetoclax [6,7,25,26]. Cell death was identified by autophagy and methuosis. The latter was originally defined in glioblastoma cells after ectopic expression of activated RAS [27]. In this context, RAS signaling is of interest because ectopic expression of an activated form of the GTPase HRAS oncoprotein (G12 V) induces massive cytoplasmic vacuolization and caspase-independent cell death in cultured glioblastoma and gastric cancer cells [28]. Methuosis is responsible for the increased rate of non-apoptotic cell death observed in our cell lines, when cisplatin and venetoclax are combined. While other mechanisms may induce methuosis in parallel with oncogenic RAS, our data suggest a more complex mechanism of action for venetoclax.

Consistent with previous findings, BCL2 expression in HNSCC specimens was associated with shorter OS and PFS [26]. This, in contrast to the questionable BCL2 specificity of venetoclax, argues in favor of this protein as a potential predictive biomarker, as immunohistochemical staining is simple, *i.e.*, either scoring as negative or positive (membranous/cytoplasmic staining in ≥30 % of tumor cells).

Some studies describe methuosis-like features upon exposure to small molecules, particularly in cells resistant to pro-apoptotic drugs [27]. The Cancer Genome Atlas profiling revealed mutations in *HRAS* in approximately 4 % of HNSCCs tested. These mutations were all detected in HPV^neg^ cases, often with inactivating *CASP8* mutations, and wild-type *TP53* [29]. Furthermore, cells with an oncogenic *RAS* were extremely sensitive to the inhibition of protein kinase C and BCL2 could antagonize this apoptotic process [7,30]. Additionally, mechanisms of resistance to venetoclax-based therapy in acute myeloid leukemia include *TP53* gene mutations or inactivation of the p53 protein, and activating kinase mutations such as RAS. Given that *TP53* mutations (a) occur in ∼ 72 % of HNSCC cases with enrichment in the HPV ^neg^ group, (b) confer cisplatin resistance, (c) are currently undruggable [31,32], and (d) can co-occur with *HRAS* mutations, venetoclax represents a realistic treatment option for a subset of HNSCC patients.

The patient of cell line HNSCC48 was suffering from recurrent oral cancer and was treated with chemoradiation after the initial diagnosis. While the initial tumor did not stain for BCL2 by immunohistochemistry, the recurrent tumor tissue taken to establish the cell line stained positive for BCL2 in 50 % of tumor cells (Supplementary Figure 1). This suggests that the BCL2 may be associated with a more aggressive phenotype. In addition, EMT is a major contributor to drug resistance and, conversely, may be driven by cisplatin through the up-regulation of Snail and N-cadherin in HNSCC cells [5,33]. Evasion of the immune response by altering the expression of molecules involved in immunosuppression or immune evasion is another important mechanism of EMT-driven resistance. Upregulation of BCL2 is associated with cisplatin resistance *via* inhibition of Bax translocation [34]. In cervical cancer, a lower dose of cisplatin is feasible when combined with BCL2 silencing as an adjuvant treatment to reduce resistance [35]. The addition of venetoclax to cisplatin reduced cisplatin-induced EMT in our cell lines. While the beneficial cytotoxic effect of BCL2 inhibition in parallel with standard therapy has been shown in HNSCC cell lines [7,25], the inhibitory potential of EMT is a novel finding. Since cisplatin increases BCL2 levels, the inhibitory function of venetoclax targets EMT independent of treatment-naïve BCL2 status. It is known that BCL2 family members regulate EMT through calcium signaling [36] and that BCL2 induces EMT in mammalian cell lines [37]. The exact interplay is complex and not fully understood. In the context of a possible broader pharmacological axis for venetoclax beyond BCL2, this agent may be integrated into cisplatin-based treatment regimens for selected patients.

This study has some limitations. First, experiments could have been performed with more cell lines. We attempted to address this by using three patient-derived cell lines from different anatomical sites, including one cell line from a recurrent cancer, each with a different molecular profile. Second, the *in vitro* part did not consider HPV-related effects, although HPV-driven HNSCC respond better to chemoradiation than their HPV-negative counterpart. As we did not have HPV-positive cell lines established in our lab, we focused on the HPV-negative counterpart, and observed no differences in the treatment effect when therapies were combined.

## Conclusion

Cisplatin is widely used to treat advanced HNSCC, but therapy resistance often occurs. Adding the BCL2 inhibitor venetoclax to chemotherapy may reinforce cisplatin-based cell death and help to overcome therapy resistance by weakening EMT and re-inducing cell death. Before this drug, which is standard of care for some hematological malignancies, can be established in HNSCC patients, biomarkers are needed to identify patients who will benefit most from a combined therapy.

## Ethics approval and consent to participate

The institutional ethic committee at the University Hospital Rostock approved the study (A2018–0003; A2022–0120). The study was performed in accordance with the Declaration of Helsinki.

## CRediT authorship contribution statement

**Anne-Sophie Becker:** Writing – review & editing, Writing – original draft, Project administration, Methodology, Formal analysis, Data curation, Conceptualization. **Friederike Klauk:** Methodology, Data curation. **Thomas Freitag:** Methodology, Data curation. **Daniel Fabian Strüder:** Writing – review & editing, Supervision, Project administration. **Björn Schneider:** Validation, Resources, Methodology, Investigation. **Annette Zimpfer:** Validation, Supervision, Resources. **Claudia Maletzki:** Writing – review & editing, Writing – original draft, Supervision, Resources, Project administration, Investigation, Formal analysis, Conceptualization.

## Declaration of competing interest

The authors declare that they have no known competing financial interests or personal relationships that could have appeared to influence the work reported in this paper.

## Appendix Supplementary materials

Image, application 1

Image, application 2
