# Supplementary material for: Inhibition of platin-induced BCL2 increase overcomes chemoresistance in squamous cell carcinoma of the head and neck through resensitization to cell death
Source: Transl Oncol. 2025 Feb 18;53:102308. doi: 10.1016/j.tranon.2025.102308 (PMC11880736; doi:10.1016/j.tranon.2025.102308)

**Supplementary Figure 1.** **Change of BCL2 immunostaining in the initial and relapsing tumor specimen from the patient, whose recurrent tumor served for establishing HNSCC cell line 48.** (A) At time point of first diagnosis, treatment-naïve tumor cells were negative for BCL2. (B) After cisplatin therapy, tumor relapsed and stained for BCL2 in about 50 % of tumor cells (200x).


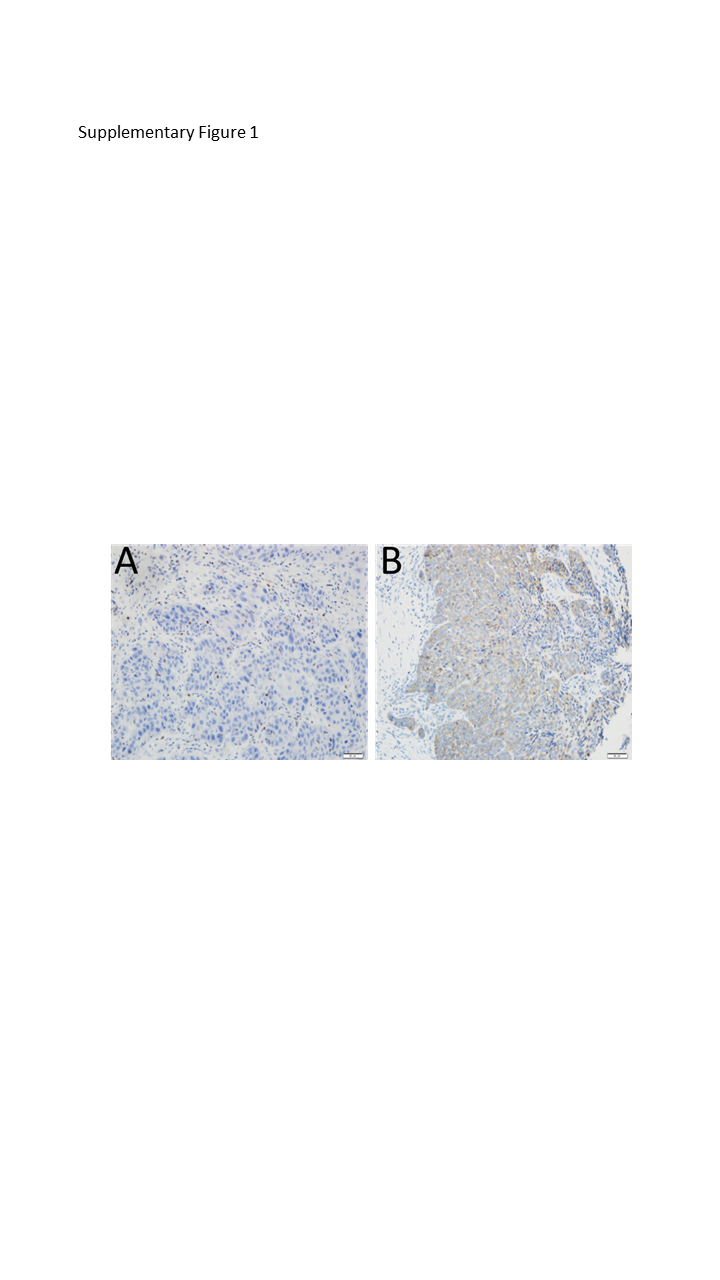

Supplement: Supplementary file 1 [file mmc1.docx]
