# Supplementary material for: Inhibition of platin-induced BCL2 increase overcomes chemoresistance in squamous cell carcinoma of the head and neck through resensitization to cell death
Source: Transl Oncol. 2025 Feb 18;53:102308. doi: 10.1016/j.tranon.2025.102308 (PMC11880736; doi:10.1016/j.tranon.2025.102308)
Supplement: Supplementary file 2 [file mmc2.docx]

Supplementary Table: Clinicopathological characteristics of the cohort (n=254):

|  |  |  |  |  |  |  |
| --- | --- | --- | --- | --- | --- | --- |
|  | **Bcl-2** | **negative** |  | **Bcl-2** | **positive** |  |
|  |  |  |  |  |  |  |
| Characteristic | No Cisplatin | + Cisplatin |  | No Cisplatin | + Cisplatin | *p-*value* |
|  |  |  |  |  |  |  |
| n | 88 | 122 |  | 16 | 28 |  |
|  |  |  |  |  |  |  |
| Median age (range) | 59,5  (36-70) | 60  (40-83) |  | 53  (45-67) | 54  (46-67) | n.s |
|  |  |  |  |  |  |  |
| Gender |  |  |  | n.s |  | n.s |
| male | 59 | 100 |  | 16 | 26 |  |
| female | 29 | 22 |  | 3 | 2 |  |
|  |  |  |  |  |  |  |
| Localization |  |  |  | n.s |  | n.s |
| Oral | 15 | 34 |  | 5 | 9 |  |
| Oropharyngeal | 43 | 41 |  | 8 | 12 |  |
| Hypopharyngeal | 8 | 22 |  | 1 | 2 |  |
| Laryngeal | 22 | 24 |  | 2 | 5 |  |
|  |  |  |  |  |  |  |
| UICC stage |  |  |  |  |  | n.s |
| I/II | 48 | 27 |  | 5 | 8 |  |
| III/IV | 40 | 95 |  | 11 | 20 |  |
|  |  |  |  |  |  |  |
| Treatment |  |  |  |  |  |  |
| surgery/  radiation only | 32 | - |  | 6 | - | n.s |
| + radiation | 56 | - |  | 10 | - |  |
| + chemotherapy | - | 60 |  | - | 15 |  |
| + chemoradiation | - | 62 |  | - | 13 |  |
|  |  |  |  |  |  |  |
| 2-year OS | 71% | 57% |  | 50% | 54% | n.s |
|  |  |  |  |  |  |  |
| Recurrence |  |  |  |  |  |  |
| Yes | 20% | 34% |  | 44% | 54% | ** |
|  |  |  |  |  |  |  |
| P16^#^ |  |  |  |  |  | n.s |
| negative | 58 | 85 |  | 12 | 20 |  |
| positive | 28 | 37 |  | 4 | 8 |  |
|  |  |  |  |  |  |  |
|  |  |  |  |  |  |  |

^*^ Significance level: ^*^ p<0.05; ^**^ p<0.01; ^#^ n=252; n.s, not significant.
